# Microplastic pollution in African countries’ water systems: a review on findings, applied methods, characteristics, impacts, and managements

**DOI:** 10.1007/s42452-021-04619-z

**Published:** 2021-05-13

**Authors:** Tadele Assefa Aragaw

**Affiliations:** Faculty of Chemical and Food Engineering, Bahir Dar Institute of Technology-Bahir Dar University, Bahir Dar, Ethiopia

**Keywords:** Abundance, Analytical techniques, African water system, Plastic pollution, Sampling methods

## Abstract

**Abstract:**

Owed to their simplicity, flexibility, lightweight, and low cost, plastics have become highly demanded in Africa as well as worldwide. However, the management of plastic wastes, particularly in African countries, is inadequate and most of the plastic debris is gatewayed into the water bodies. Nowadays, environmentalists, organizations, and governments are aware of microplastic pollution in the marine and terrestrial environment. Thus, addressing a compressive literature review in one referenced paper, as they draw up the articles, is essential to propose new research directions, to synthesize the existing theories among the existing studies. The abundance of microplastics is variable depending on the sampling and identification techniques. In this review, the available publications on microplastic pollution in African countries’ water systems were retrieved. Investigations found that microplastic pollution levels in the studied water bodies were reported in high concentrations. It was observed that different sampling and analytical methods were applied for the detection of microplastics, and suggestions were raised at it may affect the reliability of the results. Most of the detected and quantified microplastics were confirmed as they are from secondary sources. Most of the microplastic pollution research was conducted dominantly in South Africa, and secondly Nigeria, although other countries should also start conducting in their water systems. Surface water and sediment samples were dominantly carried out, but are limited with biota samples; hence, the risk assessment of microplastics is not yet determined. Some of the African countries have regulations on the prevention of macroplastic wastes, but the implementations are unsuccessful and most have not yet been established resulting in a threat of microplastics pollution. Thus, the research priorities on microplastic detection should be identified, and the African countries’ governments should be more proactive in eradicating macroplastic, which ends up as microplastics, pollutions in the water environments.

**Graphic abstract:**

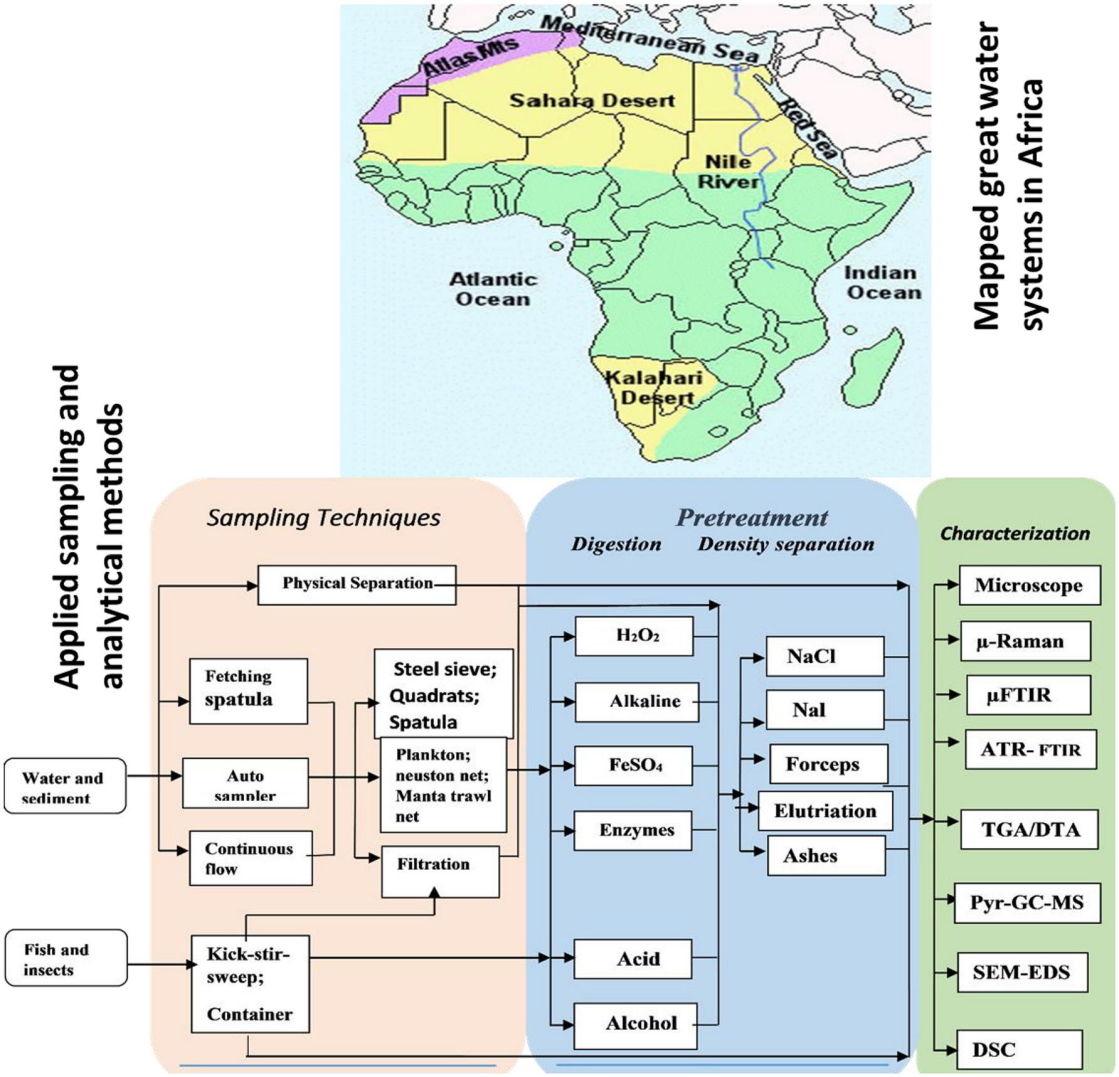

**Article Highlights:**

- Researches on microplastic pollution in African countries water system is limited .
- A high microplastics abundance is found in African countries water system.
- Sampling methods and used analytical techniques for microplastic detection were included.
- Harmonized standard methods for microplastic pollution research should be established.
- Combined analytical tools at once should be adopted to detect reliable microplastics.

## Introduction

Investigations of the microplastics (MPs) occurrence (fragmented from macroplastic wastes and primary microplastic sources manufactured as microfibers and others) were reportedly found. An estimated amount of 275 million metric tons that produced in 192 coastal countries since 2010, out of this 4.8 to 12.7 million metric tons of plastic waste ended up in the ocean, were reported (Jambeck et al. 2015). Approximately 6300 million metric tons of plastic waste had been generated globally as of 2015, and around 9 and 12% had been recycled and incinerated, respectively. The rest of the plastic wastes, around 79%, have been disposed of in landfills and/or discarded in the natural environment (Geyer et al. 2017). Not only the inadequate disposal of single-use plastics, source of MPs, but also the fabric nanofibers from the personal protective equipment (PPE), such as medical face masks and hands-on gloves, are important sources in the water systems that required attention as emerging research needs (Aragaw 2020; De-la-Torre and Aragaw 2021), such as the sources, occurrences, distribution of MPs, and the risk assessments to the aquatic biota are required as prior research. As a result, the aquatic environment is prone to become MPs sink by different transportation mechanisms.

Recently, the MPs have been recognized and attracted attention as emerging contaminants in the aquatic system. MP pollutants in the environment were first attempted in the early 1970 s and later described by marine biologists since 2004 (Thompson et al. 2004). The research output and efforts increased enormously as compared with lines of research. MPs (< 5 mm in size) may be the polymeric plastic materials that originate through fragmentation of macroplastics used in many personal care products, called secondary MP sources. In addition, the primary sources are those in which they are deliberately manufactured through extrusion or other techniques to be used as an ancestor to other products and/or direct uses are an important source for MPs pollution (Browne 2015). Commonly manufactured products of primary MPs are plastic pellets; abrasives, synthetic fibers (Hidalgo-Ruz et al. 2012). The occurrence and abundance, and quality of MPs have been investigated in different aquatic environments including sediments, and wastewaters worldwide both in the urban and remote areas. Dominantly, studies investigating MPs have mostly failed to assess the pollution in the terrestrial and soil environments. However, recent reports also considered the assessments of MPs pollution in the terrestrial and soil environments in such a way that the agrochemicals could be a potential source for the pollution of MPs (Dioses-Salinas et al. 2020). Attention has been raised by the environmentalists due to the adverse impacts of MPs on the natural environment and aquatic biota. Higher organisms (e.g., fish) and microorganisms in the aquatic system can ingest MPs and obstruct or suffer from starvation due to pseudo satiety (Cole et al. 2011). The ingested toxic chemicals leached by MPs could reach human beings through the food chain (Hermabessiere et al. 2017). Besides its impact, MPs can act as vectors for the transport of other toxic chemicals, such as Polycyclic Aromatic Hydrocarbons (PAHs), Polybrominated Diphenyl Ethers (PBDEs), and heavy metals, among others by different chemical and/or physical mechanisms (e.g., adsorbing toxic materials on the surface of MPs) (Wardrop et al. 2016). The evidence in the globe has found that humans are exposed to xenobiotic contaminated seafood through ingestion which is unavoidable and ultimately can cause a risk to food security as well as the human health problem (De-la-Torre 2020). Moreover, the spatial and temporal variations in the trophic transfer systems and mechanisms of MPs, and the potential risks to high trophic levels of animals have been reported by Farrell and Nelson (2013), Setälä et al. (2014), Shi et al. (2018).

Africa is a developing continent that needs a large demand for plastic products due to their low cost and easy availability. China is the largest plastic polymer producer which produces 31% as of 2019 out of the global plastic production, 368 million metric tons worldwide, and is the highest export destination in Africa (PlasticEurope 2020). Although Africa has been developing rapidly and consumed large amounts of plastic products in their economic development in the past few decades, there is a lack of plastic waste management practices and plastic pollution awareness among the general public. Thus, the freshwater and marine environment in Africa are at risk of MP pollution.

Research on the MPs pollution started lately in African as compared with other continents. In 2016, the first work on MP pollution in the African Great Lakes, particularly from Lake Victoria, Nile perch, and Nile tilapia was reported by Biginagwa et al. (2016). Global Sampling of Sediment from Shores for MPs accumulation study, and South African were included as one of the shores, recorded as the first report in African waster system (Browne et al. 2011). Even in some countries, like Ethiopia having the longest river (Blue Nile), Lakes, Lake Tana, and several small lakes and/or rivers in the rift valley, almost none of the researches has been conducted on MPs till now. In 2020, the first study on microplastic pollution assessments in Lake Ziway, Ethiopia, was reported by Merga et al. (2020). Recently, environmental concerns in African countries on microplastic pollution research have attracted attention, and investigation has been increasingly reported in rivers, bays, lakes, and seas. As a result, this review aimed to assess the current research on microplastic pollution in African countries aquatic environment; deliberate the sources, transport systems, and active regulations regarding MP pollution; evaluate the potential impacts of MP pollution in Africa’s aquatic system, and identify the research problems and research directives in the future. Moreover, this review could address compressive literature in one referenced paper, as they draw up the articles, is essential to propose new research directions, to synthesize the existing theories among the existing studies.

## Data acquisition strategies and treatment

A literature search was conducted in June 2020, to retrieve articles on MP pollution from the African water system. An all-inclusive materials retrieval was performed using all accessible databases, including Web of Science (https://clarivate.com/webofsciencegroup/solutions/web-of-science/), Scopus (https://www.scopus.com), and PubMed (https://pubmed.ncbi.nlm.nih.gov/) databases; and Mendeley, ResearchGate, and Google Scholar scientific sources. Search keywords included were “microplastics AND Africa,” “plastic debris AND Africa,” “plastic AND marine pollution AND Africa,” “Africa AND water bodies.” The retrieved publications, such as research articles, review articles, case studies, technical reports, short communications, and books were screened by country and type of water system, categorized based on research scopes (identification, quantification, abundance, and occurrence), and studies only in Africa’s water systems (including across countries worldwide associated with African countries’ water system), such as rivers, lakes, oceans, and gulfs and bays were selected. The impact of MPs on fauna from Africa’s water systems was also included. A potential, and detailed studies of the identification and detection of MPs in the water system, excluding editorials, pathway modeling publications, and news, were reviewed with a total of 33 articles as from Table 1, and also 1 article focusing on the policies for the reduction of single-use plastics in Africa were retrieved.

**Table 1:** The sampling, and extraction/purification, and analysis studies on microplastic pollution in Africa’s aquatic system

Country	Study area	Sample type	Sampling method	Separation/extraction	Identification	Abundance (mean value)	Reference
South Africa (include as a shoreline from the global study)	Western Cape	Sediment from sandy beaches	As per NOAA guideline	A saturated NaCl solution	FTIR	21–30 fibers per 250 Ml of sediment	Browne et al. (2011)
South Africa	KwaZulu-Natal	Sediment, surface water	By towing with conical zooplankton net (300 μm mesh). Filtered through 1000, 500, and 250 μm sieves	Hypersaturated NaCl solution	FTIR-ATR	Durban Harbor: 159.9 ± 271.2 particles per 500 ml, uMgeni and Isipingo estuaries (41.7 ± 23.0 and 47.6 ± 22.8, respectively	Naidoo et al. (2015)
South Africa	Southeastern Africa	beach sediment, surf-zone water	Manual catching of top 5 cm of beach sediment with ziplock bags. Filtered using a WP-2 type net (80 μm mesh size)	Density separation with a saturated saline solution	Microscope visualization	688.9 ± 348.2 to 3308 ± 1449 particles m^− 2^ beach sediment; 257.9 ± 53.36 to 1215 ± 276.7 particles m^− 3^ for water	Nel and Froneman (2015)
Tanzania	Mwanza	Fish (Nile perch and Nile tilapia)	Samples were collected from Mwanza harbor market, where fish are caught and sold daily	Density separation 10 M NaOH	ATR-FTIR	Perch (55%) and tilapia (35%)	Biginagwa et al. (2016)
South Africa	KwaZulu-Natal	Fish, mullet Mugil cephalus	Using a cast-net	Manual dissecting	Visual microscope	3.8 ± 4.7 particles per fish	Naidoo et al. (2016)
South Africa	Cape Town	Subsurface waters	Continuous pumping via stainless steel pipes at the keel of the ship (depth 11 m) at a flow rate of 25 m^3^/h	Filtration onto glass microfiber; Whatman (pore size 1.2 μm)	Visually under a microscope, ATR-FTIR	1.15 ± 1.45 particles m^−3^	Kanhai et al. (2017)
South Africa	–	Salt	Purchased directly from the Malaysian market	NaI, Membrane filtration, KOH (10% w/v) solution	Visually under a microscope; Raman spectroscopy	1 to 10 MPs/kg of salt	Karami et al. (2017)
South Africa	Durban	Sediment cores	Samples Collected in a canal in a gravity corer	Density separation; H_2_O_2 (_30%)	ATR-FTIR	1750 pieces/kg-dry sediment from Durban Bay	Matsuguma et al. (2017)
South Africa	KwaZulu-Natal	Beach sediment and surf-zone water	A single quadrat (50 × 50 cm), and stored in ziplock bags. Filtered with a 10 L bucket and 63 μm mesh sieve	Density separation with NaCl solution (100 g/l)	Microscope visualization	86.67 ± 48.68 to 754.7 ± 393 particles− 2 in sediments	Nel et al. (2017)
Tunisian	Northern Tunisian	Sediment	Three 0.25 m x 0.25 m quadrats were used and removed using a clean stainless steel spatula	Sodium chloride (NaCl) 140 g L^−1^ solution	FTIR-ATR	141.20 ± 25.98 to 461.25 ± 29.74 items kg^−1^ DW	Abidli et al. (2018)
South Africa	Eastern Cape of South Africa	Sediment and larvae	Hand-held nylon net (mesh size 500 μm), and stored in a ziplock bag	Saline solution (100 g L^−1^), HNO_3_ (55%)	Microscope visualization	160.1 ± 139.5 MP particles kg-1 in sediment samples 0.37 ± 0.44 and 1.12 ± 1.19 particles mg^−1^ wet weight for the summer and winter, respectively, in the larvae samples	Nel et al. (2018)
South Africa	Cape Town	Indigenous reef-building polychaete Gunnarea gaimardi	Removed manually at the desired site, and stored with a Ziploc bag	NaCl solution (100 g-l)	Visually under microscope	0.275 ± 0.215MP particles g^−1^ dry weight	(Nel and Froneman 2018)
Nigeria	Southwestern Part of Nigeria	Gastropods	By direct searching and detaching them from stone and rock substrata	KOH (10 M) and H_2_O_2_ (34.5–36.5% v/v)	µFTIR	L. varicus load per gram wet weight = 1.71 ± 0.46 g^−1^, and T. fluviatilis load per gram wet weight = 6.1 ± 1.05 g^−1^	Akindele et al. (2019)
Nigeria	Nwangele, Imo State	Water	As per the UNEP/IOC Guidelines. Grab sampling technique at the depth of 0–3 cm with a “W” shaped design	Filtration with filter paper (pore size = 11 μm)	Microscopic visualization	3,487 items/m^2^; 469 ± 153.33 items/m^2^ (downstream); 85.8 ± 174.94 items/m^2^ (midstream); 211.4 ± 109.84 items/m^2^ (upstream)	Ebere et al. (2019)
Nigeria	Legos	Surface Sediments	Using a stainless steel spoon at a depth of 1–5 cm and with a wooden quadrant (20 × 20 cm)	Direct visual inspection	Visual with a microscope, FTIR	170 ± 21, 141 ± 36, 133 ± 16, and 121 ± 38 items, respectively, for Eleko, Lekki, Alpha, and Oniru beaches	Ilechukwu et al. (2019)
South Africa	KwaZulu-Natal	Sediment	Stainless steel manta trawl with nylon (333 μm) mesh was used	Physical separation by forceps	Dissecting microscope	4.01 ± 3.28 particles/100 m^2^ for surface trawls. 5.45 ± 3.26 and 2.96 ± 2.94 particles/100 m^2^ winter and summer, respectively	(Naidoo and Glassom 2019)
South Africa	Grahamstown	Sediment	Collected directly from the open quarry on the floodplain	Ashes at 450 °C for 12 h; Saltwater (1.07 g cm^− 3^ )	Visually under microscope	495 microbeads kg^−1^ or 78.59%	Nel et al. (2019)
Tunisia	Northern Tunisian	Sediment	Three 0.25-m × 0.25-m quadrats and the sediments were removed using a clean stainless steel spatula	NaCl solution (1.13 g cm^3^)	Microscope visualization, FTIR-ATR	2340 ± 227.15 to 6920 ± 395.98 items kg^−1^ DW	Toumi et al. (2019)
South Africa	Northwest Orange-Vaal	Water	Using neuston net (mesh size of 300 μm), and bulk water sampling	NaCl solution (1.2 g cm^− 3^)	Visual microscope	bulk water: 0.23 ± 0.27 items L^−1^; net: 0.04 ± 0.16 items m^− 2^	Weideman et al. (2019)
Nigeria	Ibadan	Fish species	109 fish samples obtained directly from artisanal fishermen	NaCl (hypersaline Solution); H_2_O_2_	Fluorescence stereo zoom microscope	A total of 69.7%	Adeogun et al. (2020)
Ghana	Accra	Fish specimens	From fishing boats with random sampling techniques for every five boat landings	10 M KOH, Heating at 60 °C for 24 h	Stereo microscope visualization	40.0 ± 3.8 to 25.7 ± 1.6 MPs particles per total fish species	Adika et al. (2020)
Nigeria	Abeokuta and Osogbo	Insects as a bio-indicator	A total of 29 insects were collected using a pond net	KOH(10 M); H_2_O_2_ (34.5–36.5% v/v)	Microscope visualization; µFTIR	(43.29 ± 43.29; 62.36 ± 3.53; 291.76 ± 26.55) for the three species	Akindele et al. (2020)
South Africa	Braamfontein Spruit, Johannesburg	Water, sediment, and larvae	Direct fetching with a container for a water sample, and collected by removing of top sediment in the river for sediment 1 mm mesh size net and a kick-stir-sweep method were used for larvae	10% KOH, density separation and with NaCl (339 g l ^−1^)	Microscope visualization	Mean of 705 particles m^− 3^ in water, 53.4 particles g^−1^ WW in larvae, and 166.8 particles kg^−1^ Sediment	Dahms et al. (2020)
Nigeria	Lagos	Beaches sediment	Using a quadrat (0.5 × 0.5 × 0.2 m), and a stainless steel spoon was used to scoop the beach sand as per the standard of NOAA	Density separation with NaCl solution	Microscope visualization, ATR-FTIR	A total of 3424 particles/m^2^	Fred-Ahmadu et al. (2020)
Ethiopia	Ziway	Fish and sediments	Collected from active fishery cooperatives. Surface sediments collected using an Ekman grab sampler (HYDRO-BIOS)	Physical separation of gastrointestinal tracts; 10% KOH	*Visual and ATR-FTIR*	0.0002–385.2) mg/kg ww (35%)in Fish; and (400–124,000) particles/m^3^ in sediments	Merga et al. (2020)
South Africa	KwaZulu-Natal	Fish	Fish species collected directly in KZN, South Africa	Proteinase K digestion	ATR-FTIR	0.79 ± 1.00 MP particles per fish in the 174 samples	Naidoo et al. (2020)
Nigeria	Yenagoa	Surface water and sediments	Teflon pump through stainless steel mesh, and with a grab on the top 5 cm	NaCl (65%); NaI; and H_2_O_2_ (30% v/v)	Microscopic visualization	1004 to 8329 items m^− 3^ for the dry season and 201to 8369 items m^− 3^ for the raining season, respectively, for water; 347 to 4031 items kg^− 1^ and 507–7593 items kg^− 1^ for dry and rainy season, respectively, for sediment	Oni et al. (2020)
Egypt	Alexandria	Fish	Using fishing net with boat support. Washed by 0.2 μm filter	10% KOH solution	Microscope visualization, Diffraction scanning calorimetry (DSC)	28 to 7527 MPs fish^−1^ for different fish species	Shabaka et al. (2020)
South Africa	Cape Town	Mussel species	Random searching, and stored into Ziploc bags with ice	KOH (10% w/v)	Visual with microscope	164 (98%). Equivalently 2.33 ± 0.2 MP particles /g and 4.27 ± 0.5 MP particle /mussel	Sparks (2020)
Algeria	Northeast of Algeria with	Surface Sediments	Scraped by metal-capped glass bijoux jar (5 ml), with rubber seal equivalent with quadrats ( 0.25 m × 0.25 m)	NaCl solution (384 Gl^−1^)	Visual counting; ATR-FTIR	182.66 ± 27.32 to 649.33 ± 184.02 Kg^−1^ dry weight (DW)	Tata et al. (2020)
South Africa	Cape Town	Marine water	Neutron net (a 300 mm) trawled by a boat	NaCl (5 M); H_2_O_2_ (30% ); iron (II) sulfate (0.05 M)	Pyrolysis-GC–TOF-MS SEM–EDS, FTIR, TGA	–	Vilakati et al. (2020)
South Africa	Southern Africa	Water	Bulk and Neutron nets sampling	Density separation with NaCl solution (1.2 g L^−1^ )	*Microscope visualization, Raman spectroscopy*	2.3 ± 7.2 microfibers L^−1^ in the wet season and 1.4 ± 2.6 microfibers L^−1^ in the dry season, and 0.2 ± 0.2 fragments L^−1^	Weideman et al. (2020)

## Sampling techniques, MPs preparations, and detection methods

**Fig. 1:**
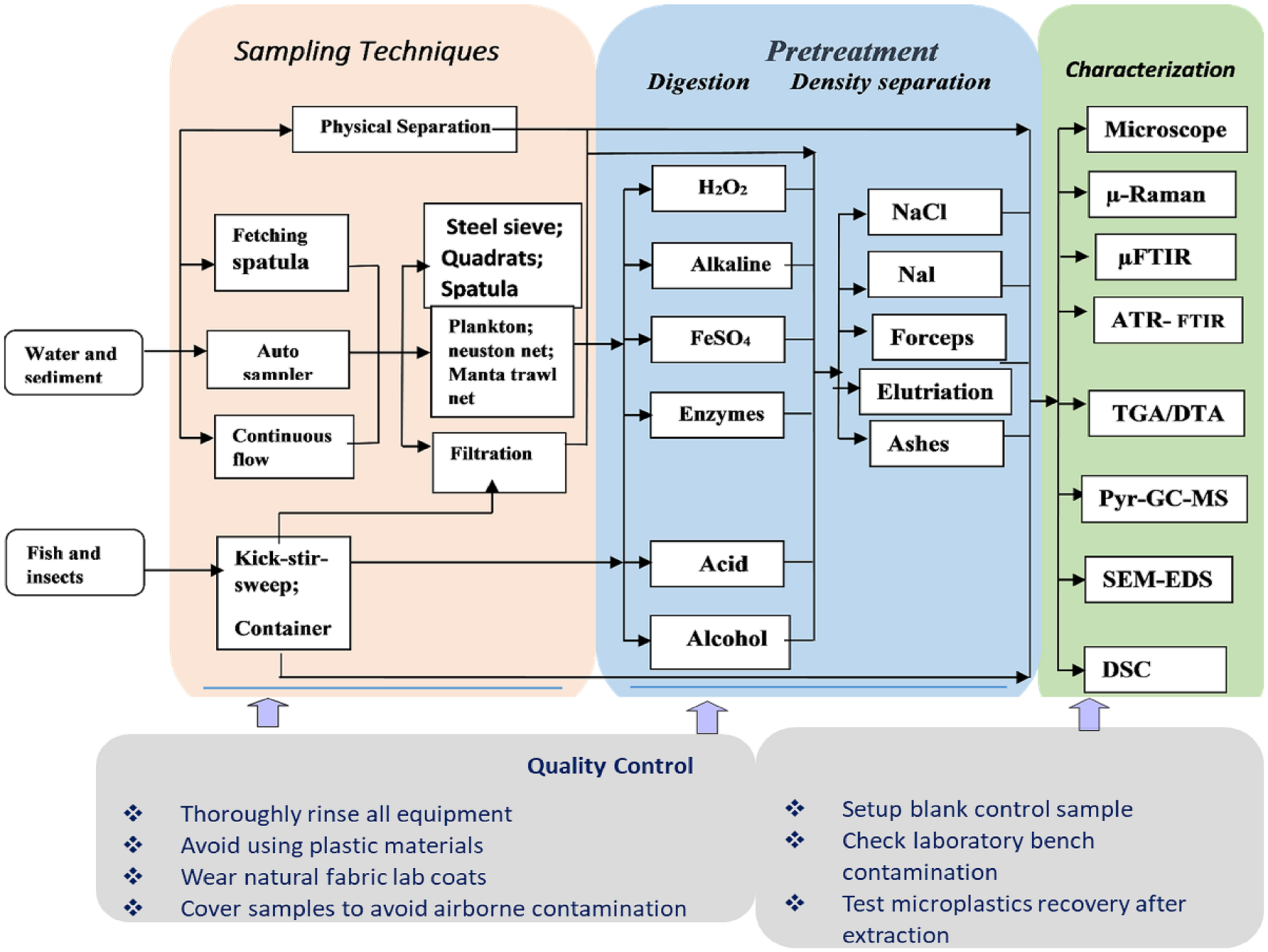
The schematic flow processes summary on the sampling, pretreatment, and identification methods used for microplastic quantification and detection in African countries’ water systems

The sampling techniques, MPs separation methods, and instrumental tools used for quantification and identification of the MPs pollution have been investigated in lakes, rivers, estuaries, and coastline in African countries’ water systems. Most of the sampling techniques and MPs preparation procedures from the retrieved publications are similar, and the flowchart that summarized the sampling, treatment, and identification methods is shown in Fig. 1. In this review, the type of samples that were studied were water, sediment, and biotic (insects and fish), and the methods used are almost similar. That is, the saturated salt solutions (with different densities) were used for the inorganic impurities separation, eventually, density separation with flotation is carried out. Different mineral acids/bases and hydrogen peroxide were used for the oxidation/digestion of organic impurities. Usually, all type of samples is collected from the water system, and physical separations (especially for the fish sample is important to separate external shells). Different instrumental analytical techniques were used, and the advantages and disadvantages of each are summarized in Table 2.

**Table 2:** The advantages and disadvantages of frequently used instrumental analytical techniques for microplastics identification Adopted from (Huppertsberg and Knepper 2018)

Methods	Instrumental Techniques used	Particle size ranges	Merits	Demerits	Remark
Visual	Microscopic (Stereo-or dissecting) Counting	The particles with size down to the µm range can be identified by the stereomicroscope	It is advantageous for a high amount of samples. Given the overall picture of microplastics abundance in a short time with low cost	It cannot determine the polymeric type and it is necessary to couple	Based on their image surface texture
Spectroscopic	µFTIR and ATR-FTIR	Plastic particle size greater than 500 μm can be analyzed by ATR-FTIR, and smaller particles less than 20 μm by µFTIR	Samples are non-destructive; Reliable data, fast and quiet acquisition of several thousand spectra	Only effective for IR-active samples; very small particle size (< 20 μm) could not have enough absorbance interpretable spectra; colorful particles are ineffective; the cost is expensive and requires skilled person; it is sensitive and can affect the environmental condition; need experienced researched for data interpretation; the sample pretreatment and background correction is mandatory	The polymeric functional group is determined
	Raman Spectroscopy	Microscopy coupled Raman Spectroscopy (RS) is suitable for 1–20 μm sized particles	Effective for small particles between 1 and 20 μm which cannot with FTIR; the resolution is high and having low sensitivity toward sample hygroscopic; effective for colored including dark particles using; chemical mapping is fast; have automatic data collection /processing feature	If the separation and purification techniques are not effective, the detection is not reliable due to high interferences of fluorescence; can hinder the identification of particles; all appropriate Raman acquisition parameters (e.g., wavelength, laser power, and photobleaching) are required; automatic mapping is still under development; time time-consuming analysis	Based on the spectral and chemical mapping
	Scanning Electron Spectroscopy	Including nano-sized particles can be analyzed	The sample is non-destructive; a high-resolution image of the samples can be produced and give reliable morphological properties of the particles	High vacuum coating of the samples is mandatory; It cannot give detailed information on the polymeric type; it is cost	Based on their surface morphology
Chromatographic	Pyrolysis GC/MS	It is suitable for only > 500 μm size samples	Organic plastic inclusive samples can be analyzed in one run without solvents which can avoid background contamination; high sensitivity and reliability; a data library is available for common polymer spectra	Particle-wise weight is measurement per run. The database is only available for selected polymers. Only effective for large size particles	Based on their molecule mass
	Liquid Chromatography	Liquidized and sufficient sample size of several milligrams is suitable regardless of particle size	Selected microplastic polymer recovery is high. Advantageous soluble plastic particles	The physical characteristics could not be investigated; Only a small amount of samples can be measured per run; Selective polymers assortment. Could not effective for non-dissolved particles	Based on the mobility (elusion) of the particle molecule
Thermogravimetric (DSC, DTA)		Independent for any particle size	Massive (Simultaneous) analysis of all-polymer type; The procedure is easy and simple	The particles are being destructed; ineffective with a few and only well-defined melting point polymers type	Based on their thermal properties (endothermic and exothermic)

### Sample techniques

Even though the studies on microplastics pollution research have been performed for years, the sampling techniques, extraction and/purification, quantification, and qualification methods are not yet standardized to date. Investigated reports from different studies worldwide as well as reviewed literature in this study are confirmed that significantly varied techniques were used and cannot be easily compared to each other. There are two sampling methods, in general, in the MPs identification of various samples. These are volume-reduced and bulk sampling techniques (Hidalgo-Ruz et al. 2012) for sediment and water samples. But, the manual gathering and boating techniques for fauna (insects, larvae, and fish species) sample ad been employed as shown in Table 1. In the volume-reduced sampling, the volume of the water in the sample has been reduced directly with direct onsite sampling, but not in the bulk sampling. “Neuston plankton net” and “manta trawl net” approaches are the two commonly used techniques for volume-reduced sampling, mostly applied for surface water sampling. As compared with volume-reduced sampling, bulk sampling techniques have not been employed for surface waters due to MPs may have a lower density than water, and floats on top of the water. Manta trawl approaches can be applied to collect surface water samples in such a way that a flow meter is used to calculate the entire volume filtered through the mesh and the total volume of water can be determined (Rocha-Santos and Duarte 2015; Free et al. 2014). Most of the studies selectively had been used nets having 0.333 or 0.350 mm mesh size, proposed by the National Oceanic and Atmospheric Administration of USA, to concentrate topwater samples mostly due to MPs could be appropriate in these rang (Arthur et al. 2009). Using the trawl approaches is advantageous due to it can cover huge sampling areas and effective volume-reduced samplings and accurate estimation of MPs can be obtained, rather than the bulk sampling techniques which can provide only a crude estimate of the number of MPs at a site. However, the trawl sampling approaches have not effective during the dry season where water levels become low, rather it is effective in the wet seasons (Weideman et al. 2020). This is due to deploying the net where the water levels high enough to be the techniques that are effective for optimum detections of MPs as a representative in the studied location. Even though different scholars worldwide have been used different mesh sizes, such as 0.080 mm by Galgani et al. (2013), and 0.450-mm mesh size (Dris et al. 2015), a greater difference in the abundance of MPs can always be observed. In this review, the different abundance of MPs was observed by using the nylon mesh size of 0.333 mm (Naidoo and Glassom 2019), nylon net mesh size of 0.5 mm (Nel et al. 2018), the neuston net with the mesh size of 0.3 mm (Vilakati et al. 2020), neuston net with a mesh size of 0.300 mm (Ilechukwu et al. 2019), zooplankton net with a 0.3-mm mesh size (Naidoo et al. 2015) as shown from Table 1. Most of the other studies used in this review were not defined as the mesh size of the nets yet. Thus, the harmonized sampling techniques and defined mesh size attracting researchers’ attention for findings to compare the data credibility and reliability. In addition to the common manta trawl methods, bulk water sampling approaches are also used. Many of the reports worldwide on the volumes of water samples in the bulk sampling techniques are compared as high as 100 L (Song et al. 2014), but, there are limited reports with 100 mL and 2 L volumes by Dubaish and Liebezeit (2013), Leslie et al. (2017), and the results are significantly different. In this review, 100 L of bulk water samples (Dahms et al. 2020), 188.692 L water samples (Nel and Froneman 2015) were reported and had a significant difference in the MPs occurrence, sizes, and type of polymers. Even some of the reports used a continuous intake of pumping from the water system to a laboratory with defined flow rates (Kanhai et al. 2017). In general, the MP particles distribution, abundance, particle length, and polymeric types and compositions (PS, PE, PP, etc.) are highly dependent on sampling techniques. Even, it is not proper to apply the same sampling techniques for the different aquatic MPs (ocean, seas, reservoirs, lake, and river). From this, it can be deduced that varied values of the mean abundance of MPs found in the water system may not be representative to the studied locations, and an urgent standard sampling technique development for water sampling I required.

### Sample extraction and separation

Before identification, and quantifications of MPs, extraction and/ or purifications for impurity removal are a critical step. This is very important to separate the unwanted matrix surrounding and/or within the MPs resulted in the sample quantifications and qualifications become reliable. Because, most, MPs density is lower than the water density, separation with density difference is a frequently used approach. Mixing of the sample of interest with the defined density saturated solutions is mostly employed. Saturated sodium chloride (NaCl) is commonly used due to its low cost and negligible toxicity to humans, and the environment (Browne et al. 2011; Claessens et al. 2011). In this review, almost all of the reports were used saturated NaCl solutions for the density separation of MP particles, floats on top of the surface, as shown in Table 1. Depending on the type of MPs constituent polymer type (such as polyvinyl chloride, polyoxymethylene, and others) that they have higher density, the saturated NaCl solution cannot be appropriate. Density separation techniques have the capability for the low-density MP particles to float to the surface of the water by settling down the coarse (e.g., clay, sand, silts), and high-density materials. As a result, the floated MPs could be taken out for subsequent experimentation. Even though there are its own hazardous, economic affordability, and separation efficiency issue, different studies by using other salts which have high density have been found for density separation of MP particles, such as sodium polytungstate (SPT) solution having a density of 1.4–1.5 kg/L (Corcoran 2015), calcium chloride having a density value of 1.3 kg/L (Stolte et al. 2015), and sodium iodide (NaI) solutions having a density of 1.8 kg/L (Nuelle et al. 2014). The separation approach by the combination of fluidization with flotation techniques after vigorous stirring is effective, and 80 to 100% of MP particles yield could be obtained (Ivleva et al. 2017). Among the different strategies for efficient separations of MPs, fluidization by NaCl solution with subsequent floatation with other high-density salt solution (e.g., NaI) is by far an effective recovery technique (Nuelle et al. 2014).

Besides the salt solution separation approaches, another important, and effective instrumental separation technique (especially efficient for larger particles in sediment samples), namely Munich Plastic Sediment Separator, has developed. This instrument has been working in combination with the use of the ZnCl_2_ solution which has a recovery rate of up to 96 to 100% (Imhof et al. 2012). Another apparatus called elutriation and floatation apparatus, which is important for the separation techniques have been reportedly used. The elutriation technique is effective (efficiency of 94 to 98%) for the separations of MPs from the sediment samples in the aquatic environment (Claessens et al. 2013). Also, the elutriation processes have their drawbacks which are inefficient MPs separations in the wastewater due to high concentrations of organic contaminants in that the density difference between the MPs and other particles within the matrices is insignificant.

Another important process after extraction for the effective identification and analysis of MPs is the oxidation and/or digestion process. The organic removal processes can be categorized into chemical degradation and enzymatic degradation. These processes have been used to remove interfering organic tissues and inorganic dust also. In this review, the degradation of impurities in the samples was found oxidation with potassium hydroxide (KOH) (Sparks 2020; Adika et al. 2020; Shabaka et al. 2020; Dahms et al. 2020), hydrogen peroxide (H_2_O_2_) (Adeogun et al. 2020; Oni et al. 2020; Vilakati et al. 2020), and sodium hydroxide (NaOH) (Biginagwa et al. 2016). KOH and H_2_O_2_ are most popular and reportedly found in the retrieved literature as well as worldwide used for effective treatment processes as an oxidizing agent. This is due to having negligible drawbacks for the damage of MP particles. Another report found that a combination of different chemicals; hydrogen peroxide with alkaline base (H_2_O_2_ with KOH) were used for the oxidation of impurities (Akindele et al. 2020), and the purification of MP particles from the matrix (Tata et al. 2020) in different samples. A critical selection of the oxidizing agents is an important issue for the effective detection of all the MP particles and components that only degrades the organic matter without degrading plastics. Reports confirm that different mineral acids such as HNO_3_ and HCl as strong oxidizing agents were used to digest the biological tissues of fish and shellfish but may destroy and/or damage the chemical and structural characteristics of MPs at low pH values (Cole et al. 2014). Ended, scholars recommended that the 10% of KOH solution is an effective alkaline treatment agent for the digestions of biological tissues without degrading the polymers (Dehaut et al. 2016). Particularly, polymers synthesized from the amide monomers, for example, polyamide plastics can be destructed and degraded with HNO_3_ solutions. In this review, one report is found thatHNO_3_ was used for the digestion process for the separation of MPs (Nel et al. 2018). Conversely, hydrochloric acid which is non-oxidizing acids are inefficient for the digestion process at low concentrations, and temperature and huge organic residue could be produced also. Another important approach to remove or reduce the organic matter is enzymatic digestion processes. The samples containing MPs can be incubated with a single and/or mixtures of enzymes, such as lipase, amylase, proteinase, chitinase, and cellulose, with the corresponding protein, lipid organics (Cole et al. 2014), and carbohydrate organics (Löder et al. 2015). Enzymatic degradation could remove high percentage organic materials, especially in plankton-rich samples. Particularly, the seawater samples are required with enzymatic digestion capable within a few hrs. In this review, one report was found that the use of proteinase K enzymatic for the digestion organic materials during the MPs separation (Naidoo et al. 2020).

Another digestion process is by using the combinations of ultra-sonication with a salt solution, particularly sodium dodecyl sulfate (SDS) (Enders et al. 2015). But, there is one critical limitation in this process. This is, the tiny MPs could be generated from the plastic samples, particularly plastic which has low elasticity properties, i.e., easily brittle plastic materials (Bergmann et al. 2016). It can be concluded that each approach during the separations of MPs for different samples has its pros and cons. Therefore, the combination of different purification processes should still be used for the maximum percentage recovery of MPs, and some optimization in the sample purification still needs to be further investigated.

### Identification and detection techniques

In this review, different instrumental analytical techniques, dominantly Fourier transform infrared spectroscope (FTIR), and microscopic visualization was carried out for the identification and detections of MP polymeric types as shown in Table 1. Only one report was found that thermal technique (differential scanning calorimetric, DSC) had been employed for the identifications of polymer types (Shabaka et al. 2020). Interestingly, one study had used a combination of different instrumental techniques for effective quantification and qualifications of MPs from the marine water samples (Vilakati et al. 2020). This study was used chromatographic-mass spectrometry (pyrolysis GC-MS) techniques for the determinations of pyrolytic polymer products, and scanning electron microscopy coupled with energy-dispersive X-ray spectroscopy (SEM–EDS) to analyze the morphological and elemental distribution of MP particles, thermogravimetric analysis (TGA) for the determination of thermal fragmentation properties, and FTIR for functional group spectral analysis.

Analysis and/or quantification of MPs is conducted after different debris, planktons, and other organic or inorganic matrices are purified. The physical methods of identification by visual categorization based on their size, shape, and color are the first important steps. This is very important to select which instrumental techniques is appropriate for identification and quantification of them based on their polymer types (Rochman et al. 2015), particle sizes, and structural feature (Qiu et al. 2016). But, microscopic visualization accuracy depends on the extraction, and purification techniques, because other similar particles with their size and shape (e.g., planktons, inorganic clays) may be counting as MP particles. Thus, other identification techniques, spectroscopic, and microscopic technical approaches are mandatory. Especially, for smaller MP particles, instrumental tools for effective identification are required. Identifications of MPs by visual sorting could be affected by several factors, such as personal errors, the counting device quality resulted in approximately 70% of error rates could be observed (Hidalgo-Ruz et al. 2012). Recently, investigative research on MPs analysis reportedly uses chromatographic, and spectroscopic approaches as effective quantifications, and qualifications techniques. Among the widely used instrumentals tools: (1) Chromatographic techniques (Nuelle et al. 2014), gas chromatography-mass spectrometry (GC-MS) in the pyrolysis working process (Hintersteiner et al. 2015), and liquid chromatography (Elert et al. 2017). (2) Spectroscopic techniques (Ivleva et al. 2017); Raman spectroscopy (Zhao et al. 2017), and FTIR spectroscopy (Imhof et al. 2016). Each instrumental techniques have their limitations and merits (Qiu et al. 2016). The most popularly used instrumental analytical techniques with their merits and demerits are provided as shown in Table 2. Particularly, micro-Fourier transforms infrared spectroscopy (µFTIR) and Attenuated total reflection-Fourier transform infrared spectroscopy (ATR-FTIR) is very important, and frequently used due to the ability of simultaneous visualization, mapping, and collections of IR spectra (Wiesheu et al. 2016).

## The occurrence of microplastics in African countries aquatic systems

The abundance of MPs can be quantified with different units depending on the sample and sampling types. For example, surface water, sediments, and biota samples can be quantified as items/L, items/kg, and items per individual, respectively (Yuan et al. 2019). Sometimes, it can be enumerated with a percentage (Vilakati et al. 2020). County-wise, 7 publications from Nigeria water bodies next to South Africa (18 publications) were found and retrieved in this review. The very limited researches on the MPs pollution in the water system, one publication for each, was found from Tunisia, Tanzania, Egypt, Algeria, Ghana, and Ethiopia. Almost all other countries in Africa have not yet conducted MPs pollution research on their water systems including Ethiopia which has the biggest Lake and River (Lake Tana and Blue Nile River). In this review, dominantly the gulf, bays, and coastline of oceans and/or seas and Harbor location in the water system researches were reportedly investigated. Reports only in three Lakes, Ox-Bow Lake, Eleyele Lake, and Lake Victoria were found.

### The occurrence of microplastics in lakes

The great lakes have been formed at the end of the glacial period 14, 000 years ago in such a way that the ice sheet is treated and exposed to the basins which had been carved into the land, eventually filled out with melted. Mostly, there are shallow areas that could be up-flow filled and drained by groundwater based on groundwater balance. Also, rainfall and the nearby rivers are sources for Lake waters. Thus, the source of plastic waste in the Lake is come from runoffs, rivers, and municipal and industrial effluents, and accumulated from the surface water as well as the sediments depending on the plastic densities. In African Countries’ lakes, MPs pollution has been reported in Lake Eleyele from Nigeria, Lake Victoria from the coast of Tanzania, Oxbow Lake from Nigeria urban lakes, and Lake Ziway from Ethiopia. In Oxbow Lake, the abundance is significantly varied from 1004 to 8329 items m^− 3^ and 201 to 8369 items m^− 3^ for the dry and rainy season, respectively, in surface water with different sampling sites. From the surface sediment, 347 to 4031 items kg^−1^ and 507 to 7593 items kg^−1^ were recorded in the dry and rainy season, respectively (Oni et al. 2020). In Lake Ziway, the abundance was found with varied range across different sampling zones (0.0002–385.2 mg/k WW in fish), and (400–124,000 particles/m^3^) in sediment samples (Merga et al. 2020). These varied ranges are attributed to the varied feed sources of the plastics to the Lake. The seasonal variations in the MPs abundance arise due to the lack of proper sewage management and unselective dumping around the site, and also, the farming and fishing activities which contributes a significant variation. Based on the sample site perspective, the sites where the boat station near the coast have been observed higher MP abundance as compared with other sites. Also, the MPs distribution where the site near the lakeshore was detected in high abundance is due to recreational tourism-related plastic goods that could contribute a huge abundance (Zhang et al. 2018). In conclusion, the wide ranges in MP abundance detected in different sample types could be due to human activities, waste management practices, geographic location of sampling sites. Moreover, it could be due to the sampling techniques, and detection methods.

### The occurrence of microplastics in rivers

Rivers, large or small, are natural streams that flow through a channel in the surface of the ground, eventually goes into other water bodies (ocean, lakes, sea, rivers, and sometimes into groundwater). As compared with other water systems, rivers, are important transporters of wastes, in the present case plastic wastes, and they are the most important feeder of MPs into the marine water system (Lebreton et al. 2017). Quantification and identification of MPs had been studied in ten different rivers with different geographical regions from South Africa and Nigeria. Ogun and Osun rivers from Nigeria; Obiaraedu, Nwangele, Okumpi, Ogbajarajara, and Onuezuze Rivers from southeastern Nigeria; Jukskei River, Orange-Vaal River, Bloukrans River from South Africa were studied. MP pollution in River Jukskei from water and sediment samples at the urban streams have been studied and recorded different values in different sampling sites. The mean values of MPs are recorded as 705 particles m^− 3^ in water, and 166.8 particles kg^−1^ in sediment samples (Dahms et al. 2020). The MP pollution from the Rivers Obiaraedu, Nwangele, Okumpi, Ogbajarajara, and Onuezuze from southeastern Nigeria was reported in one research article. The MP abundance were recorded as 3487 items/m^2^; 469 ± 153.33 items/m^2^ (downstream); 85.8 ± 174.94 items/m^2^ (midstream); 211.4 ± 109.84 items/m^2^ (upstream) (Ebere et al. 2019). The highest abundance of MP downstream could be the feed small rivers carrier plastic wastes and end up at the downstream of the big river where the samples were taken. Average MP abundance was recorded as 1.7 ± 5.1 L^−1^ equivalently > 99% fibers in Orange-Vaal River, south African water samples independent of sites, and confirmed that the river is highly polluted (Weideman et al. 2020). The MP abundance in Bloukrans River, South Africa from sediment samples was potentially studied in other rivers. The mean abundances were recorded as 160.1 ± 139.5 MP particles kg^−1^ in sediment samples independent of the season (Nel et al. 2018). In general, all the studied samples from all geographical areas river waters system confirmed that highly polluted with MP particles.

### The occurrence of microplastics in ocean/sea coastlines and bays

The coastlines, sometimes called seashores are the lines that form the boundary between the land and the ocean/sea. The oceans are not freshwater, rather marine environments that are highly important for shipping transportation. The well-known and biggest seas and oceans in Africa are the Mediterranean Sea, and the Atlantic Ocean, respectively. Sea and oceans are the highest-end receptors of plastic debris that comes from other water bodies (lakes, rivers, ponds, and reservoirs. In this review, dominantly the identification and quantification of MPs are reported from the bays and coastlines of seas/oceans. Twenty types of research out of 32 publications on the MPs pollution research in the sea/ocean coastline, estuary, gulf, and bays have been reported as illustrated in Table 1. Three studies of bays, gulfs, and coastlines from the Mediterranean Sea in Tunisian (Shabaka et al. 2020), Egypt, and Algeria coast were first reported (Tata et al. 2020). The identification of MPs pollution from sediment samples on the Tunisian coast in the Mediterranean Sea was first reported (Abidli et al. 2018). The abundance of microplastic in the sediment was found in varied ranges (141.20 ± 25.98 to 461.25 ± 29.74 items kg^−1^ DW) in different sampling sites. The occurrence of MPs in this study is for the first time on the Tunisian coast of the Mediterranean Sea and results showed that extensively polluted. In Algeria, the occurrence and characterizations of MPs from the North African coasts of the Mediterranean Sea were studied (Tata et al. 2020). The varied range on MPs abundances was recorded as 182.66 ± 27.32 to 649.33 ± 184.02 kg^−1^ dry weight (DW) in different sampling locations. The MPs pollution in the Gulf and Bay of sediment Core samples from the Asia and Africa border have been identified (Matsuguma et al. 2017). The mean abundance was found as 1750 pieces/kg-dry in the sediment core. The plastic debris and the corresponding MPs have been identified from urban estuaries of KwaZulu-Natal, South Africa in different sampling zones (Naidoo et al. 2015). The mean average abundance was found at 745.4 ± 129.7 particles per 500 ml. The highest abundance was recorded in the Bayhead area, and the smallest plastic concentration was found at sampling sites away from the city center. A quantitative analysis of MPs from sediment and water samples on the southeastern coastline of South Africa has been reported (Nel and Froneman 2015). The abundance was recorded in a range from 688.9 ± 348.2 to 3308 ± 1449 particles m^− 2^ for sediment samples, and 257.9 ± 53.36 to 1215 ± 276.7 particles m^− 3^ for water samples. The presence of MPs is governed by the water system circulation, not associated with the proximity of land use and/or the population density. Three studies of MPs occurrence in sediment samples from the tropical Atlantic Ocean, Gulf of Guinea, Nigeria (Fred-Ahmadu et al. 2020), in the Atlantic Ocean seashore, South Africa; Atlantic Ocean bays across Germany, and South Africa were first reported (Vilakati et al. 2020). The first studies on the distribution and characterization of MPs pollution in sediment samples were reported in the Atlantic Ocean, Nigeria (Gulf of Guinea) (Fred-Ahmadu et al. 2020), and a range of MPs abundance was recorded as 3.4 ± 3.5–173 ± 21.3 MPs /kg for the different beaches. Significantly varied MPs abundance in different beaches may be due to the different tidal waterlines (high and drift) systems. The MPs abundance in the Atlantic Ocean seashore was not quantified, which needs quantitative and occurrences studies(Vilakati et al. 2020). However, the abundance was quantified as a mean average of 1.15 ± 1.45 particles m^− 3^ in the Atlantic Ocean bays around South Africa in the subsurface water samples (Kanhai et al. 2017). The other studies on the MPs occurrence were carried out from the coastline, gulfs, bays, and estuaries with detailed physicochemical characterizations, the study location, and abundance are illustrated in Tables 1 and 4. In general, the present author (s) concludes that the MPs pollution research with water and sediment samples from the urban areas are highly polluted as compared with the locations face by city centers. However, their abundance/concentration recorded was the highest in the sediment samples as going far from the city at which land-based plastic pollution is low.

### The occurrence of microplastics in wastewater

Wastewater treatment plants, especially municipal wastewater treatment plants (WWTPs), are channels through which secondary microplastics (MPs) are released into aquatic environments. From African countries’ WWTPs, there are no studies found, as of June 2020, on the identification and detections of MPs both in sewage and industrial effluents. However, worldwide reports, even if the studies are limited, confirm that the huge secondary MPs have been released from the WWTPs to the natural water bodies. Some of the examples are: reports from Canadian municipal WWTP confirmed the annual MP items have been entering into WWTP records that 1.76 ± 0.31 trillion. Of these, 1.28 ± 0.54 trillion are from primary sludge, 0.36 ± 0.22 trillion from activated sludge, and 0.03 ± 0.01 trillion are discharged into the water environment (Gies et al. 2018). Other reports from 12 WWTPs in Germany a total of 9*107 to 4*109 MP particles discharged annually efflux tested WWTP, and the authors recommend tertiary wastewater treatment unit processes as a post-filtration that can reduce total MP discharge by 97% (Mintenig et al. 2017). Microplastics concentrations in sewage sludge in Sweden, USA, Scotland, Netherlands Finland, China, Ireland, and Germany were detected as 720 ± 112 (Magnusson and Norén 2014), 4000–5000 (Carr et al. 2016), 1200–7868 (Murphy et al. 2016), 660–760 (Leslie et al. 2017), 23,000–170,900 (DW) (Lares et al. 2018), 1565–56,386 (DW) (Li et al. 2018), 4196–15,385 (DW) (Mahon et al. 2017), and 1000–24,000 (DW) (Mintenig et al. 2017), respectively. Because wastewater treatment plants (WWTPs) are an important pathway of MPs entering into natural aquatic systems, the present author recommends that effective detection of MPs and understanding of the abundance and fate from African countries WWTPs should be researched out.

### The occurrence of microplastics in fauna from all studied African’s water systems

Reports in Lake Eleyele, Osun River, Lake Victoria, Jukskei River, Bloukrans River, the Eastern Harbor of Mediterranean Coast, Eastern seashore Ghana, Durban Harbor estuaries, Bloukrans River of Grahamstown, and Osun River were investigated MPs occurrence in different gastropods, insects, larvae, immobile warm species in the tube structure, and fish species samples. The MPs abundance, physical and chemical properties detected from water systems of biota samples. The different larvae species samples, with varied feeding habits, have a similar abundance even though the samples were collected from different African countries and water systems. This suggests that almost all biotas can ingest MP particles in their habitat systems. MPs pollution in African countries’ water bodies, dominantly with different South African provinces were studied in several gulfs, bays, lakes, and estuaries, and their abundance is varied. The aquatic biotas are recommended as they are an indicator of MPs pollution level of the water bodies due to their correlation with the water and sediment MPs pollution level as well. This was confirmed by Dahms et al., in which MPs from the three different samples (water, sediment, and larvae species) at the same sampling area were detected and have their correlation (Dahms et al. 2020). The MPs in Lake Eleyele were detected from a total of 109 fish samples consisting of eight species and were averagely found as a total of 69.7%. The different abundance is due to the maturity, different organs, and ingestion capacity of the species (Adeogun et al. 2020). The highest proportion was observed in the stomach. The abundance of MPs from Lake Victoria was studied by taking two different fish species (Nile perch and Nile tilapia), and was detected from the gastrointestinal tracts and observed 55% from 11 perch and 35% from 7 tilapia. The different abundance in those species is due to their feeding preference of the specific properties (Biginagwa et al. 2016). In the Ogun and Osun rivers, the MP abundance in different insect samples was observed as 43.29 ± 43.29; 62.36 ± 3.53; 291.76 ± 26.55 particles/species for the three different species (Akindele et al. 2020). MP observed with varied order of insects is due to different feeding tells than aquatic faunas of different taxonomic and /or ecological niches could be likely on MP pollutants. The abundance of MP in the Chironomus sp. is significantly higher as compared with the other two. This is due to the high ability to gather and depositing organic materials (Nel et al. 2018). MP pollution in River Jukskei from larvae species samples at the urban streams have been studied and recorded different values in different sampling sites. The mean values of MPs are recorded as 53.4 particles g^−1^ in larvae species samples (Dahms et al. 2020). The MP abundance in Bloukrans River, South Africa, from larvae samples was potentially studied as compared with the other rivers in the country. The mean abundances were recorded as 0.37 ± 0.44 and 1.12 ± 1.19 particles mg^−1^ wet weight for the summer and winter, respectively, in the larvae species (Nel et al. 2018). In this study, the abundance of larvae species was observed 75% in the summer season, but the occurrence increased to 98% in the winter season. From this, it can be concluded that the larvae species studied are significantly seasonal dependents. Other reports confirmed that the important MPs identification studies in fish samples were carried out in Eastern Harbor from the Mediterranean Coast of Egypt (Shabaka et al. 2020). A range of abundance with the three different species was recorded as 7527, 3593, and 1450 MPs fish^−1^. The varied MPs abundance is observed based on the fish species plastic ingestion ability, and the accumulation capacity which corresponds with the weight and length of the species used. Thus, it can be deduced that the African countries’ water system is polluted by MPs, which has been affecting the aquatic animals. However, the present author has not found any report of MPs’ occurrence and impacts on the aquatic plants from the African countries’ water system.

### Worldwide microplastics in the water system

MPs are passed and get into the world’s water system through coastline, beaches, bay, and gulfs in runoff sediments from the littering. The abundance of MPs from some countries worldwide, other than Africa, retrieved literature is reviewed and illustrated in Table 3. The sample type and quantified concentration MPs in different locations of freshwater and marine environment were compared. Some of the retrieved literature selected and used for comparison of MPs abundance is based on, intentionally, the different geographical areas, not more than one or two articles in a country, and studied locations to be the review inclusion across a country. Different reports confirmed that studies were carried out by using various sampling techniques separation procedures, and instrumental methods, and the quantity and quality of the detected MPs are also varied. The MPs abundance and/or concentration can be expressed in different units of measurement depending on the techniques used. Established standard techniques for effective (Filella 2015), and reliable determination (Hidalgo-Ruz et al. 2012) of MPs have been recommended by many researchers including the present author (s). Thus, direct comparisons of the microplastic abundance are not reliable due to the methods used in the studies could be varied (Qiu et al. 2016). However, the reports possibly provide an overview of insights into MPs abundance with their pollution levels. As can be seen, MPs quantified are much higher in some countries than others. Comparatively, the abundance in ocean water is higher than the freshwater systems, such as 466,000 particles km^− 2^ MP particles concentrations were detected from the Laurentian Great Lake water system (Eriksen et al. 2013). Thus, it can be concluded that the MPs concentration from freshwater may indicate the current pollution level, but the cumulative pollutions could be reflected from the marine environment. Studies from the Easter Island Chile, Danish waters from Denmark and Jurujuba Cove and Niterói, RJFive urban from Brazil reported that the small abundance/concentration of MPs was recorded as 0.072 g/L, 0.0324 g/L, and 0.099 g/L, respectively, as compared with other countries.

**Table 3:** Comparisons of microplastics abundance and studied locations in aquatic systems worldwide Modified from (Li et al. 2020b)

Country	Location (Studied water)	Sample type	Concentration	Estimated MP units L^−1^	References
UK	Kelvin River	Sediment	0.26685 g/L	296.5	Blair et al. (2019)
China	Poyang Lake	Sediment and Surface water	0.2034 g/L	226	Yuan et al. (2019)
Europe	Carpathian basin	Sediment and surface water	0.4716 g/L	524	Bordós et al. (2019)
China	Wei river	Sediment and surface water	0.918 g/L	1020	Ding et al. (2019)
Belgium	Flemish rivers	Water	0.0153 g/L	17	Slootmaekers et al. (2019)
Australia	Bloukrans River	Sediment	0.216 g/L	240	Nel et al. (2018)
Malaysia	Surface water in Malaysia	Surface water	0.108 g/L	120	Praveena et al. (2018)
Canada	Lake Winnipeg	Surface water	1.7397 g/L	1933	Anderson et al. (2017)
India	Vembanad Lake	Sediment	0.27 g/L	300	Sruthy and Ramasamy (2017)
Italy	Lake Chiusi and Lake Bolsena	Sediment and surface water	2.5 particles/m^3^	0.025	Fischer et al. (2016)
Brazil	Jurujuba Cove, Niterói, RJ Five urban	Sediment and surface water	0.099 g/L	110	Castro et al. (2016)
France	River Seine, urban area	River water	3 particles/m^3^	0.03	Dris et al. (2015)
Mongolia	Lake Hovsgol	Lake water	1.2 × 10^4^ particles/km^3^	0.00012	Free et al. (2014)
Chile	Easter Island	Sediment and surface water	0.072 g/L	80	Hidalgo-Ruz and Thiel (2013)
South Korea	Heungnam beach	Sediment and surface water	0.3285 g/L	365	Heo et al. (2013)
Denmark	Danish waters	Sediment	0.0324 g/L	36	Strand et al. (2013)
USA	Great Lakes	Surface water	1.6 × 10^7^ particles/km^3^	0.016	Eriksen et al. (2013)
Switzerland	Various lakes	Sediment and surface water	2 × 10^3^ particles/m^3^	20	Faure et al. (2012)

*Sea salts are not classified into water bodies (salt sample used for MPs identification were taken from commercial markets).

Styrene ethylene butylene styrene (SEBS), acrylonitrile butadiene styrene (ABS), chlorinated polyethylene (PE-C), polypropylene (PP), and Polystyrene (PS), polyurethane (PU), polyethylene terephthalate (PET), polyethylene-polypropylene copolymer (PEP), Ethylene propylene (EP), polyacrylates, Cellulose triacetate (CTA), Poly (Ethylene Vinyl Acetate) (PEVA), low-density polyethylene (LDPE), high-density polyethylene (HDPE), and syndiotactic polypropylene (sPP), Polyvinyl Chloride (PVC), polyamide (PA), polyacrylic acid (PAA) and ethyl vinyl acetate (EVA), Polyisoprene (PI)Polyacrylonitrile (PAN), Polyethylene/polypropylene copolymer (PE/PP cop), silicone rubber (SR), styrene acrylate (SA), polymethylmethacrylate (PMMA), Cellophane (CPH), acrylic resin (ACR).

## Physical and structural characteristics of microplastics in the African water system

MP particles have no unique characteristics, rather they are found in different physical, chemical, and structural properties. Depending on the virgin plastic sources, MPs have been found with different densities, shapes, colors, lengths, and polymer types. This may arise also from their additives contained contaminants within the plastic polymer. The MP particle characteristics have a direct correlation with their toxicity (Frère et al. 2017). For example, among different types of MP shapes, fibers are confirmed that the most toxic for biotas in the water system (Ziajahromi et al. 2017). The light weightiness, length, and polymeric shape highly affect the floating/ sediment down nature of particles (Kaiser et al. 2017). In this review, the basic characteristics of identified MPs in African countries’ water systems, from the retrieved kinds of literature, are adequately analyzed in the studies. Some of the studies were not carried out the necessary properties of MP particles. The essential properties (shape, color, polymer type, and size), and study locations of identified MPs in African countries’ water systems are illustrated as shown in Table 4.

**Table 4:** The physical and chemical Characteristics of microplastics reported in Africa’s water bodies

Studied water system	Size/length	Shape of MPs	Color	Polymer type	References
Beaches	–	Dominantly fibers	–	PS (56%), acrylic (23%), PP (7%), PE (6%), and PA (3%)	Browne et al. (2011)
estuaries (Durban coastline and beaches)	250 to 500 μm	pellets, fragmented, films, scrubbers, monofilament line, twine, and fibers	–	Dominantly PS	Naidoo et al. (2015)
Southeastern coastline	0.065 to 5 mm	Dominantly fibers and fragments	Blue, black, green, and red	Dominantly PS	(Nel and Froneman 2015)
The southern shore of Lake Victoria	< 0.5 mm	–	–	PE, PU, PS, PP, PEP and silicone rubber	Biginagwa et al. (2016)
Durban Harbor	0.2 to 15 mm	Fibers (51.2%), Fragments (34.6%), polystyrene (7.3%), films (5.0%), monofilament line (1.5%) and twine (0.4%)	White (41.8%), clear (22.0%), opaque (13.2%) and black (5.5%) for fragments, and other types (17.5%)	–	Naidoo et al. (2016)
Bays, from the Atlantic Ocean	0.25 to 5 mm	Predominant fibers (94%)	Blue (72%), transparent (9%), pink (8%), and others (11%): purple, brown, red, green, gray, black, yellow, and white	PS (49%), blends of PA or acrylic/polyester (43%), and others (8%): PP, acrylic, PVC, PET, and PU	Kanhai et al. (2017)
*Sea salts from different countries	160 to 980 µ m	Fragments (63.8%) followed by filaments (25.6%), and films (10.6%)	–	PP (40.0%), PE (33.3%), PET (6.66%), polyisoprene/polystyrene (6.66%), PAN (10.0%), and PA-6 (3.33%)	Karami et al. (2017)
Gulf and Durban Bay	315 μm–1 mm	Fibers, fragments Pellets, films, and foams	Black, white, blue, purple, red, green, yellow, gray, and transparent. Dominantly (about 84% of the total)	PE, PP, PS, PET, PEP, and PAK	Matsuguma et al. (2017)
Richard’s Bay Harbor and Durban Harbor	63 to 5000 μm	Fiber, fragment, nurdle	Black, black gray, light gray	–	Nel et al. (2017)
Tunisian coast of the Mediterranean Sea	0.1 to 5mm	Fibers, fragments, Styrofoam, pellets, and films	Black > transparent > white > red > blue > green for fibers, blue > white > clear > red > green > yellow > black for fragments, blue > white > black > clear for films while only white pellets and Styrofoam	PE, PP, PS	Abidli et al. (2018)
the coast of South Africa	–	–	–	–	(Nel and Froneman 2018)
Bloukrans River in the eastern periphery of the Mediterranean Sea	2–5 mm	–	–	–	Nel et al. (2018)
coastal KwaZulu-Natal	–	Dominantly fragments (23.3 to 72.7%), fibers (2.3–43.3%), and film (10.8–33.3%)	Main white, clear, opaque, blue, and black in winter and clear, green, and pink colors in summer were found	Only PS were detected	(Naidoo and Glassom 2019)
Bloukrans River	Large; ~1000 μm and small; <400 μm	–	White (27 ± 25.5%), and green (76.5 ± 18.2%) microbeads for the larger size. White and green microbeads of 5.5 ± 2.5% and 8.6 ± 6.0%, respectively, for small size	–	Nel et al. (2019)
Osun River	–	Fiber and film	Back, blue, brown	PE, PP, nylon	Akindele et al. (2019)
Obiaraedu, Nwangele, Okumpi, Ogbajarajara, and Onuezuze Rivers	Approximately 11 μm	Dominantly Fragments. Fiber and film in small proportion, and others	–	PET (29%), PE (22%), PVC (16%), PP (14%), and others (6%)	Ebere et al. (2019)
beaches (Alpha, Oniru, Eleko, and Lekki)	–	Dominantly fragments. Pellets, and fibers in small proportional	–	PP, PE, and PS	Ilechukwu et al. (2019)
Streams around the lagoon	0.2 to 5 mm	Fragments (42.86%) as PE, and 57.14% as PP. films (50%) as PE and 50% as PP	Black, transparent, white, red, blue, green, yellow for fibers White, blue, black, red for fragments and red, white, clear, green, blue, black for films	Dominantly PP, PE	Toumi et al. (2019)
Orange–Vaal River dams	–	Fibers (98%) and fragments (2%) in the bulk sample. Hard plastic pieces (85%) others (15%) for neutron net samples	Blue (92%), red (7%), purple (< 1%), transparent (< 1%), green (< 1%) and pink (< 1%) for fibers. Blue (38%), white (25%), orange (25%) and green (13%) for bulk samples transparent (39%), white (35%) and blue (15%) transparent (45%) for neutron net samples	–	Weideman et al. (2019)
Eleyele Lake	124 μm-1.53 mm	–	–	–	Adeogun et al. (2020)
Central Atlantic Ocean, Coast of Ghana	19.0 to 28.0 cm	Pellets (31%), microbeads (29%), burnt film plastics (22%), clear plastic fragment (6%), white plastic fragment (3%), thread plastics (2%) and microfibers (2%)	White, green, and clear	–	Adika et al. (2020)
Ogun and Osun rivers	–	Fibers; fragments	–	ABS, PE, PP, and PS for Chironomus sp.; PS, ABS for Siphlonurus sp. PS, PP FOR, and L. Viridis	Akindele et al. (2020)
Urban stream	53-4000 μm	Filaments (76.3%), round (6.3%), angular ( 2.9%) and other shaped objects (14.3%) for water samples; Filaments (95.0%), angular (3.6%), round (0.4%), other shapes (1.0%) for larvae filaments (19%), round (11%), angular (0.9%) and (68%) for sediment samples	Transparent white (30%), blue (29%), black (21%) and others for water sample; Blue (37.4%), black (23.5%), red (13.2%), transparent white (3.9%), green (2.9%) and other colors (19.0%), for larva samples; Transparent/white (80%), black (7.9%), blue (5.7%), green (0.3%) and others (4.9%) for sediment samples	–	Dahms et al. (2020)
The Gulf of Guinea from the tropical Atlantic Ocean	1–5 mm	5% pellets, 33% foam fragments, 4% fibers and 58% hard fragments	white, pink, green, black, blue, clear (transparent), and yellow	PE, PP, PVC, PA, PS, PU, EVA, ABS, and PET	Fred-Ahmadu et al. (2020)
Lake Ziway	0.15-40 mm	Pellets and fibers	–	Dominantly PP, PE, and alkyd-varnish. PU, PS, PE/PP cop, PET, SR, SA, PMMA, PA, CPH, and ACR in a small proportion	Merga et al. (2020)
East coast of South Africa	0.1 to 4.8 mm, averaging 0.89 ± 0.77 mm	Fibers (68%), fragments (21%), and others (11%)	Dominantly blue. Other in a small proportion	Rayon (70.4%), PS (10.4%), nylon (5.2%), PVC (3.0%), and other (11%)	Naidoo et al. (2020)
Oxbow Lake	0.02–5 mm for both season	Fiber, beads, fragment, pellet, films, flakes	black, yellow, green, red, blue, white, and purples	For the dry season, PET and Plasticized PVC 63% in water and 10.9% in sediment. For raining season Plasticized PV 81.5% and LDPE 4.2%, respectively	Oni et al. (2020)
Eastern Harbor from Mediterranean Coast	25 to 1000 μm	Filaments, Foam, fragments ( sheet-like fragments, Colored fragments)	–	PEVA, LLDPE, HDPE, PET, and sPP	Shabaka et al. (2020)
the west coast of Melkbosstrand	50 and 1000 μm	Filaments (67% ), fragments (21% ), and spheres (12% )	Black/gray (37%), blue/green30%), white (12%), transparent (11%), red/pink (8%), and yellow/orange (3%)	–	(Sparks 2020)
Gulf of Annaba in coasts of the Mediterranean Sea	0.81 to 2.16 mm	fibers (70%), fragments (21%), pellets (5%), films (2%) and foams (2%)	black, white, blue, purple, red, green, yellow, gray, and transparent	PE (48%), PP (16%), PET (14%), PS (9%), butyl Branham (7%), EP (3%) and CTA (3%)	Tata et al. (2020)
bays and beaches of Atlantic Ocean seashores	–	–	–	Dominantly PE (85.7%), PET (71.4%) PVC (57.1%). PS, PA, PAA, and EVA are with a small proportion	Vilakati et al. (2020)
Orange and Vaal River	20 − 5,000 μm	Dominantly Microfibers (97.2%), fragments (2.8%): 43% for green, 25% for white, 17% for blue, 9% for yellow, 3% for orange and 3% for pink/red	Dominantly Blue. Green, white, yellow, orange, and pink in a small proportion	–	Weideman et al. (2020)

From the 25 retrieved publications, out of 33 works of literature, the size of MPs was determined, and the other 8 studies were not determined the length of the MP particle as shown in Table 4. The different size distribution was observed, and different techniques were adopted by different scholars. Averagely, the largest MP length abundance was found in the seashore of Ghana (Adika et al. 2020). The next large-sized MP abundance was identified from the southeastern coastline of South Africa (Nel and Froneman 2015). The small-sized ranged from 0.011 to 0.5315 mm MP abundance was found in the river water bodies; Obiaraedu, Nwangele, Okumpi, Ogbajarajara, and Onuezuze Rivers (Ebere et al. 2019), Bloukrans River in the eastern periphery of the Mediterranean Sea (Nel et al. 2018), Bloukrans River (Nel et al. 2019). The size of MP abundance from the three studied lakes, Oxbow Lake (Biginagwa et al. 2016), Eleyele Lake (Adeogun et al. 2020), and Victoria Lake (Oni et al. 2020) have been recorded as 2.41, 0.827, and < 0.5 mm, respectively. More or less, the estuaries, coastlines, bays, and gulfs of the Mediterranean Sea and the Atlantic Ocean have been observed that a similar length of MP abundance ranged from 0.63 to 2.6 mm. The MP sizes recorded are dependent on the sampling techniques, pretreatment methods, and identification techniques used. Also, the water systems (freshwater and marine water), the sample type (sediment, water, and biotas), and the sampling site (polluted and less polluted areas) have fundamental effects. Mostly, MP with low density could float on the top surface of the water, and would not able to detect in the sediment samples. The high-density MP is vice versa to this. Thus, harmonized standard methods for the detection of MPs are required to report viable size distribution.

Identifying the shapes of polymeric characteristics is very important for the subsequent policy establishments on plastic waste management. Because the shape of the MPs detected has a direct indication of the virgin macroplastics. In this review, the identified shapes of MPs were found from 28 articles as shown in Table 4. Some of the scholars have also quantified the shapes in percentages. Generally, the shape characteristics of the MP were classified as fragments, filaments, fibers, film, pellets, sheets, beads, flake, angular, round, and foam. Dominantly, the fibers and fragment shapes of MP abundance were observed for all types of samples. The fiber MPs are considered as pollution could contribute from the fabric (textiles) pathways, and films are from single-use plastic bags and packaging plastic materials (Kanhai et al. 2017). However other infrequent shapes, such as pellets, irregular shapes, spheres are originated from different commercial goods and transportations, especially occurred in the municipality (Fred-Ahmadu et al. 2020). The high percentage of filaments, fragments, and fibers particularly were recorded within fish (Adeogun et al. 2020), larvae (Akindele et al. 2020), and insect (Dahms et al. 2020) samples. Spheres and sheet MP percentage abundant were observed in a small proportion as compared with other shapes. As a concluding remark, the MP particles detected in African countries’ water systems are mostly from secondary MP sources, as per the present author (s) knowledge and also the evidence from the highly recorded shapes found as fragments, films, and fibers.

The color of the MPs detected is another important characteristic of virgin plastics. In this review, the twenty reports were found that the detected MPs were recorded the color of particles, and most of them are quantified in percentage constituents also. MPs having transparent colors are basically from the packaging materials which are single-use products. However, the black, blue, red yellow, and other colors are originated from resistant packaging materials, and also from other plastic goods. As shown in Table 4, a high percentage of transparent detected MPs, approximately 80%, was observed from Jukskei River from sediment samples, but, 30% for water samples and 3.9% for larvae samples (Dahms et al. 2020). In this review, generally, the color (black, blue, and red) MPs, especially in the urban estuaries, gulfs and bays were found more abundantly. Many of the reports from the retrieved literature confirmed that different color MPs in different locations and sample types in the same water system were found.

In addition to the physical characteristics of MPs, the chemical composition (polymer type) is crucial to ascertain a certain conclusion to the virgin plastic wastes. In this review, twenty-one studies were found in African countries’ water systems that contain the polymer type information. Dominantly, Polypropylene (PP), polyethylene terephthalate (PET), polystyrene (PS), and polyethylene (PE) types of MPs were found. This validates that those dominant polymeric plastic types are the most widely used products for commercial good in African countries which accounts for the high abundance of MPs. The reason why also PS was found dominantly is due to the consumer product protecting plastics (for example foam packaging peanuts for shipping, food packaging, meat/poultry trays, and egg cartons) is widely used in the studied area. This is because those materials are advantageous as compared with other plastic polymers for protection against damage or spoilage of foods (Naidoo and Glassom 2019).

## Effects and potential risks of microplastics in water systems

Many studies on the impacts of plastic wastes as macroplastics and their fragments called MPs on different water systems have been investigated. Particularly, in addition to human, the environmental deterioration (Auta et al. 2017), the impacts on aquatic organisms (Chae and An 2017) have been reviewed. Among the different effects of MPs on the aquatic organisms, scrapes, and abscesses, clogs of the digestive tract have been mostly reported. These effects have a direct impact on the organisms in such a way that the high mortality rates, decreased fertility, the provocative response on their biological systems, changes of metabolism mechanisms, modifications on their nourishing system, and reproductive disturbance. Besides, the organisms could be impacted by the release of toxic monomers and/or additives during the degradation of plastic polymers. MPs could be the vectors for other associated contaminants by adsorbing them on them and could have more effects on the organisms. Because MPs can adsorb heavy metals, and complex chemicals would be formed in the water system (Aragaw and Mekonnen 2021a). Li et al. also reported that MPs extracted from sewage sludge could be used as potential adsorbents for Cd, Pb, and Co removal which is an indication of they can act as a vector in the natural environment (Li et al. 2019). The effects of these MPs have been influenced by the physical (particle size, shape, and color) of the structure of the particles, and the chemical structure (polymer types), such as PE, PP, and PS. Even though the degree of their impact is varied, all reports have been shown that they have negative effects for both the aquatic organisms and in the environment itself. Some researchers and industrialists have been used microbeads (primary MPs sources) for ecotoxicity studies as a model, and they cannot put any sayings on the effects of morphological features of sampled MPs in the open environment. In this regard, many scholars have worried and expressed their fillings about current practices of ecotoxicological studies of MPs (Connors et al. 2017). Thus, they have pointed out the need for refining the reliability (Lambert et al. 2017), and the significance of extensive research on MPs (Lenz et al. 2016).

In addition to the impacts on aquatic organisms, the environmental effects of plastic waste and, their fragmented MPs have been reported in a good manner (Rochman 2016). Waste plastic bags, cans, and bottles have been disposed of on the littering and interring into the water bodies together with the sediments making anoxic conditions within it which could reduce the productivity, and lowering of the abundances of invertebrates (Green et al. 2015). This could make the disappearance of biodiversity within a long period, and the final will disturb the total ecosystem balance. Even some of the polymers (e.g., polyester) have an impact on different bacterial biochemical constituents in the aquatic ecosystem (Kesy et al. 2016). Also, the MP exposure to the water system could have an impact on the natural filtration of mechanism, and decrease the nutrient concentrations (e.g., ammonium, phosphate, calcium, magnesium, etc.) results in a decrease in biomass growth, such as cyanobacteria and green algae (Green et al. 2017). Thus, the totality of ecological degradation and deterioration by MPs is being the future direction to be investigated to be an input in the mitigation efforts for the organization, and policymakers (Rochman 2016). The effect of MPs studies is not associated directly to perform the environmental risk assessments so far. This is due to the inconsistent results obtained on the field-collected MPs sample, and the MP samples used in the ecotoxicological investigation. The risks of MPs based on the abundance, they are relatively low investigations have been carried out in the aquatic systems. Because MPs can act as niches for organisms (Aragaw and Mekonnen 2021a), the physicochemical, and morphological features of their complex niches should be critically studied in the future to investigate the ecotoxicological impacts as compared with the simple MPs only (Yu et al. 2020). Other than the MPs, nanofibers, and fabrics as a nano-plastic material has not been researched out and monitored. This may be a tie to inappropriate and/or standardized sampling techniques and analysis methods (Henry et al. 2019). The nanofiber is more toxic than the macroplastics most of which are produced by the electrospinning fabrication processes. The toxicity that is produced is due to the charging and melting in the fabrication processes. One of the recent concerns on nano-plastic fabrics is surgical face masks which have been fabricated in a huge amount to control COVID-19 pandemics. Some researches confirmed that the presence of nano-plastics in the nanofiber in personal care products (Schwaferts et al. 2019). Thus, researches on the nano-plastics including surgical face masks by developing effective sampling, and analytical methods are essential to identify and quantify within the different environments (aquatic, sediment, soil, etc.) including their fate and impacts on the fauna and flora in the future.

## Plastic management and regulation in Africa and other countries

MPs are originated from plastic polymers solid wastes, also sometimes from wastewater. Intentionally also from microfibers and microbeads being a source of primary MP particles. Thus, managing the plastic wastes following solid waste regulation is indirectly managing the MPs in particular. There are many sustainable options for plastic wastes management, such as recycling, recovery, and reusing. Depending on the polymeric type, the plastic wastes can be converted into fuels via pyrolysis, and other technologies including the surges of medical waste. Aragaw and Mekonnen (2021a, b) reported that fuels can be produced from polypropylene and polyvinyl chlorides plastic polymers, such as single-use face masks and surgical gloves that are an important source of MPs recently (Aragaw and Mekonnen 2021b). Generally, the management practices of solid wastes are implemented under Environmental Programs worldwide. Particularly in Africa, the solid wastes are managed under Environmental Protection Organs as a general, the Authority which constitutes the Council, the Sectorial and Regional environmental units and agencies mentioned under different Articles and Proclamations, with a defined proclamation number. The proclamation number and establishment years are different for the different countries in Africa, such as the proclamation in the federal democratic republic of Ethiopia was provided for the establishment of environmental protection organs since 2009 with a proclamation number of 295/2002. Recently, most of all African countries have updated the environmental protection organs into two separate organizations as “Environmental development and management,” and “environmental protection, regulations, and monitoring.” This was intentionally proposed for the sustainable use of the environmental resource, and effective management, thereby avoiding possible conflicts of interests and duplication of efforts. Also, it was necessary to establish a system that fosters coordinated but differentiated responsibilities among environmental protection agencies at federal and regional levels. Some of the countries have been managing the solid waste under the urban administration authorities in the regional cities, and some are under environmental protection agencies, specifically, Solid Waste Management Proclamation (SWMP) is established. Most of the African countries have established this proclamation recently. For example, the federal democratic republic of Ethiopia has established since 2014. In this proclamation, solid waste is well categorized as glass containers and tin cans; plastic bags; used tries; food waste; hazardous waste; construction debris. The general obligation, management planning, transportation systems, disposal cite constructions, audition, civil liability, penalty, and effective date on the solid waste are properly stated in this law including a detailed description of each section, but the implementation is limited still. In addition to the solid waste proclamation laws, other legislation under the water, and energy minister, and Lakes protection and development agency (for example established in 2020 in Ethiopia) have a contribution to the prevention of marine environment pollution.

Some African countries, especially Western African countries are increasingly adopting single-use plastic pollution reduction policies even though the policies have been limited implementation and analysis. The policy instruments used are the legislative of single-use plastic bag bans. Eleven countries have instituted bans, 1 country has a market-based instrument and the other 4 countries have no strategy out of 16 Western African countries (Adam et al. 2020). Those countries that have bans legislative carry good punishment proclamations, such as penalties and prison sentences. But, the legislation has a limited consultation during the ban drafting stage which lacks important provisions, such as reusable alternatives, the national campaigns are not yet established, and poor enforcement. Also, the ban announcement to the public is not notified as early as possible before the subsequent implementation. Thus, the present author (s) suggests that the policy revisions by including the stakeholders, consultants with enough time between announcement and implementation, and including cost-effective reusable alternatives should be made.

Besides the African countries’ laws and regulations, worldwide regulations have been established and implemented on the prevention, and management of plastic wastes. Example The People’s Republic of China has established different provisions on the solid waste management system including the plastic bag ban systems. The State Economic and Trade Commission (SETC) announced the immediate cessation of the production and use of disposable foam plastic tableware (SETC 2001). The General Office of the State Council (GOSC) limited the production, sale, and use of plastic shopping bags; banned ultrathin plastic bags (b 0.025 mm), and implemented charges for the use of other plastic bags in market places beginning on June 1, 2008 (GOSC 2007a). Also, the GOSC promulgated a new regulation on waste import (GOSC 2007b). In this regulation, importing the 24 different types of waste have been banned, including plastic waste derived from daily use. Other regulations on the Management Methods of Import of Solid Waste, and Electronic Waste. Another important and recent regulation named the National Oceanic and Atmospheric Administration (NOAA) under their project on the marine waste actions acts approved by the European Commission (Li et al. 2020a). The project is specifically established for awareness creation through the public education program toward plastic particle pollution in the marine environment.

As stated above, several proclamations, laws, and regulations on solid waste management systems, and specifically plastic waste are already established in African countries. But, the management practice on these rules is negligible so far, even not yet totally considered in some countries. Unlawful discarding of solid wastes and littering of the cities and rural areas and the water systems is uncontrolled in many African countries. The present author (s) have been observing a huge plastic waste in the marine environment of many African countries which ends up as MPs pollution and due to the unimplemented regulations. Because almost all African countries are listed under the low-income categories, the peoples have been forced to use low-cost and very thin plastic products in their small markets for different personal goods. Next to textile fabrics, plastic industries are emerging, and extensively used factories in Africa, which is not yet regulated due to the intense rest of the economy. Up to date, using all of the plastic products in person and/or at the organization level is allowed in African countries without regulation implementation. Thus, the governments of African countries need to be more hands-on in preventing plastic pollution problems to prevent environmental contamination as well as human health as responsible bodies.

## Conclusions, recommendations, and future directives

MPs pollution has been a recent emerging threat in the aquatic ecosystems. Pollutions and their toxicity has been reported widely worldwide. Even if scholars conducted so far on the MPs pollution in African countries’ water systems are limited, the available literature indicates that MPs pollution is ubiquitous in the assessed water environments. Researched water bodies located in African countries are seriously polluted, with a huge MPs abundance. However, there is limited awareness and knowledge about MPs in the water systems such as detailed impacts on biotas subsequently to human health, environmental deterioration, and fast plastic management practice. This review synthesized the current practice and status of MPs pollution and future research directives in the aquatic environment in African countries’ water systems. The sampling methods, identification techniques, occurrences, and abundance of MPs were discussed. Also, the effect and risks of MPs in the water system, and plastic waste regulation were assessed. The basic sampling techniques during MPs identification: volume-reduced, and bulk sampling techniques were described together with their advantages and disadvantages to offer for scholars with the choice of suitability to use. Illustrations on the MP particles separation and purification techniques from different samples were stated. The retrieved literature confirmed that the analysis and identification with visualization methods (microscopy counting), spectroscopy techniques (FTIR, Raman spectroscopy, and scanning electron spectroscopy), and chromatographic techniques (pyrolysis-GC/MS, and liquid chromatography) were employed, and this review discussed the pros and cons of them. The previous reports on the identification and detection of MPs, almost all, were used spectroscopic techniques, especially the attenuated total reflection-Fourier transform infrared (ATR-FTIR), and micro-Fourier transform infrared (µ FTIR).

The poor management and inadequate enforcement of relevant laws and regulations have led to the current status of MP pollution in African countries’ water systems. In this review, the author (s) found that there are no adequate researches conducted on MPs pollution from African countries’ water systems, such as no research has been conducted on the big Lake, Lake Tana, and River, Nile River, (the world’s largest river); even only one report was conducted and found so far on the worlds’ 3rd largest Lake, Lake Victoria. Thus, extensive researches on the quantification and detection of MPs from the African aquatic system is required together with the macroplastic waste management practice. Also, it has been seen that quantification and identification of MPs using a single analytical technique and sampling methods, which could not give the entire quantity and quality of the particles; hence, a combination of techniques is recommended. The combinations of different techniques can improve peoples’ understanding of MPs pollution, and standardized sampling methods are required for more reliable data worldwide.

In African countries aquatic environment, the following issues of priority should be addressed:

- Standardized and optimized sampling and analytical methods for the reliability of MPs pollution.
- Investigation on the transport, and environmental conditions for the degradation mechanisms of macroplastics, and impacts of MPs in aquatic biota and fauna is required.
- Development and pathway models for the assessment of MP accumulation in dynamic water systems and input lines to the oceans should be researched.
- Evaluation of the impacts of MPs on the socio-economy and environment is needed.
- Investigating the effect of MPs on the organisms from the molecular to community levels, and the associated contaminant influence on the ecosystem and their toxic effects should be conducted.
- Development of rules and regulations, based on the life cycle assessment (LCA), for the production of biodegradable plastic products as substituents of fuel-based plastics, is required.

Monitoring, research project, management, and policy-formulation are required that will help the researchers for a better understanding of the risks of MPs pollution in water systems and used as a guide for the development of plastic waste management systems and plans. African countries’ governments should formulate plastic waste management practices, strengthen the enforcement of relevant laws and regulations, establishing policies, and increase publicity and education to improve people’s awareness and knowledge of MPs pollution effects on the environment and the organisms as well.
